# A Case of Chronic Wernicke’s Encephalopathy: A Neuropsychological Study

**DOI:** 10.3389/fpsyt.2014.00059

**Published:** 2014-05-27

**Authors:** Erik Oudman, Stefan Van der Stigchel, Albert Postma, Jan W. Wijnia, Tanja C. W. Nijboer

**Affiliations:** ^1^Department of Experimental Psychology, Helmholtz Institute, Utrecht University, Utrecht, Netherlands; ^2^Slingedael Korsakoff Center, Rotterdam, Netherlands; ^3^Department of Neurology, Brain Center Rudolf Magnus, University Medical Center Utrecht, Utrecht, Netherlands; ^4^Brain Center Rudolf Magnus, Center of Excellence for Rehabilitation Medicine, De Hoogstraat Rehabilitation, University Medical Center Utrecht, Utrecht, Netherlands

**Keywords:** Wernicke’s encephalopathy, Korsakoff’s syndrome, confusion, delirium, dementia, amnestic, cognitive disorders, thiamine

## Abstract

A 54-year-old woman was referred to our Korsakoff Center because of extensive cognitive problems following acute Wernicke’s encephalopathy (WE). She had a relatively short history of alcohol abuse and was found lying on the floor in her home by her son. After 5 days without treatment, she was diagnosed with WE in a general hospital. During the course of the disease, minimal change to the acute situation occurred, with chronic confusion, attention deficits, and incoherent behavior symptoms most notable unlike classical Korsakoff’s syndrome. Neuropsychological assessment after 4 and 16 months after admission to the hospital revealed global cognitive decline, with striking impairments in attentional, executive, and memory functions. The present case study suggests that the state of confusion and the neuropsychological symptoms in WE can become chronic in case of very late treatment. We therefore recommend that confused alcoholics should receive appropriate parenteral thiamine according to the current clinical standards.

## Introduction

**Figure 1:**
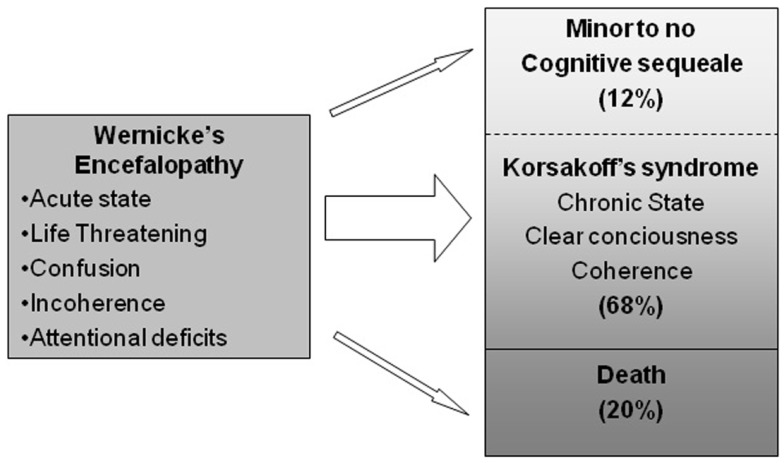
**Evolution of Wernicke’s encephalopathy as proposed by Harper et al. (3) and Day et al. (11)**. See Kopelman et al. (8) for a review.

Wernicke’s encephalopathy (WE) is an acute neuropsychiatric syndrome resulting from vitamin B1 (thiamine) deficiency (1). The syndrome is characterized by confusion, attentional disorders, incoherence, eye-movement disorders, and ataxia, although frequently only one or two characteristics are present (2–4). In the industrialized world, most patients with WE have a background of chronic alcoholism and self-neglect (5). WE requires immediate treatment with intravenous or intramuscular thiamine. When patients with WE are promptly treated with parenteral thiamine replacement therapy, this is a life-saving measure that also may prevent the development of chronic brain damage (6). When WE is left untreated or is inappropriately treated with either low doses of thiamine or oral thiamine replacement therapy, this may result in a life-threatening situation with mortality rates up to 20% of the patients (4). In the patients who survive with this lack of treatment, varying degrees of brain damage develop, although the exact course of illness is not well-understood (7). Progression into a well-known form of chronic amnesia, Korsakoff’s syndrome (KS), is not uncommon [Ref. (8), see Figure 1]. Usually, KS follows WE when acute confusion improves within one to a couple of weeks. Primary characteristics of this improvement include the ability to concentrate for a longer time, less incoherence in behavior, and clear consciousness over the course of the day despite severe amnesia (8–10).

Descriptions of cases of WE where the symptoms do not improve rapidly are scarce and often restricted to the acute situation. Examples of such cases have been described in the course of World War II following starvation in prisoners of war (12). Descriptions of the cognitive sequelae in the course of prolonged confusion following WE are currently not available in the literature. Such neuropsychiatric descriptions of severe cases of WE are crucial, however, as it will give insight in the severity of the syndrome. Despite the current standards of emergency care in the industrialized world, a vast majority of patients with WE are inadequately managed, resulting in patients who show confusion for a prolonged time (13–15). Here, we present a case study that illustrates the cognitive and behavioral characteristics, including signs of confusion, of a patient who was admitted to our clinic with untreated WE. Neuropsychological assessment revealed striking cognitive impairments unlike KS. Importantly, the initial cognitive and neuropsychiatric problems did not resolve in time, providing an example of a patient with acute WE that became chronic.

## Case Report

### Biographical history

According to members of the family, the patient functioned independently prior to the neurological problems. The son of the patient visited her several times a week. In her house she was able to manage the household, despite her alcohol abuse. The amount of alcohol she consumed varied between one and three bottles of wine each day. She divorced 12 months prior to diagnosis with WE. Up until 6 months before admission, the patient worked in a reel kitchen in a hospital. The last month, she had not been eating well and consumed alcohol throughout the day. He told that the patient did not feel like eating, but still spoke about things she wanted to undertake to manage her alcoholism. The day before she was found, she had a telephone call with her son. The son reported that the telephone call included a regular exchange of information without any striking exchange of information.

### Medical history

Our patient is a 54-year-old woman with a 7-year history of alcohol abuse. The patient did not have a psychiatric history. She was found in her house on the floor of the living room. According to reports of her son who found her, she was unable to move her legs, showed odd eye movements, had visual hallucinations of food lying around her, talked nonsense, and was incontinent of urine. After a visit of a general practitioner to her house, her son was told to give his mother rest. After 5 days and no apparent change to her situation, a visiting psychiatrist referred her to the hospital where she was diagnosed with WE and underwent a therapy of oral thiamine treatment. Subsequently, the patient was admitted to a department of an addiction center where she stayed for almost 8 months. Here, her behavior was described as incoherent and confused. She showed a compulsion to visit the toilet regularly and to frequently ask the nursing staff for cigarettes. After her stay in the addiction center, she was referred to our clinic for patients with KS.

## Methods

Four months after she was found, a neuropsychological evaluation was performed (T1) in the addiction center. Sixteen months after the first evaluation, the cognitive functions were re-evaluated (T2). Results of the first and second neuropsychological evaluation are displayed in Table 1. A chronic KS reference group is displayed in the last column. The results for the reference group were adopted from group studies on chronic KS patients as displayed in Table 1. The first neuropsychological evaluation consisted of the Wechsler Adult Intelligence Scale-III (16), the Raven Standard Progressive Matrices (17), Word-Fluency Animals and Professions (18), Digit Span Forward and Backward (16), Rey’s Complex Figure Task (19), Wechsler Memory Scale-R, Hooper Visual Integration (20), Stroop (21), Trail Making Task (22), and the Behavioral Assessment of Dysexecutive Syndrome Key Search Task. The second neuropsychological assessment consisted of the Mini Mental State Examination, the Cognitive Screening Task (23), Word-Fluency Animals, Digit Span Forward and Backward, Trail Making Task, the Visual Association Test (24), Rey’s Complex Figure Task and the Frontotemporal Dementia Rating Scale (25). The patient was informed by the first author and asked whether she was willing to participate. Written informed consent was obtained from the legal representative. The procedure was performed in accordance with the guidelines of the Declaration of Helsinki and guidelines for recruitment of incompetent patients [cf. Ref. (26)].

**Table 1:** **Test results of neuropsychological assessment after 4 and 16 months after admission to the hospital in our case and a KS reference group**.

	Case: neuropsychological assessment 1 – 4 months after admission			Case: neuropsychological assessment 2 – 16 months after admission			
Cognitive domain	Test	Patient	Patient compared to KS reference group	Test	Patient	Patient compared to KS reference group	KS reference group
Orientation	Time and place (MOCA)	2**	*z* = −1.1	Time and place (MOCA)	2**	*z* = −1.1	3.6 (1.5)^a^
Global cognitive functioning				MMSE^¶^	21/30**	*z* = −0.6	22.8 (3)^b^
				Cognitive Screening Test^¶^	10/20**		N.A.
Language	Word-Fluency Animals (1 min)^¶^	15**	*z* = −1.1	Word-Fluency Animals (1 min)^¶^	10**	*z* = −1.3	36 (20)^c^
	Word-Fluency Professions (1 min)^¶^	13**	*z* = −1.5				35 (15)^c^
Working memory	Digit span^¶^	5*	*z* = −3.9	Digit span^¶^	8*	*z* = 0.4	7.7 (0.7)^b^
Long-term memory	Rey’s complex figure recall^¶^	0**	*z* = −0.9	Rey’s complex figure recall^§^	0**	*z* = −0.9	2.5 (2.7)^d^
	WMS-R logic memory^¶^	7**					6 (N.A.)^e^
	WMS-R visual reproduction^¶^	0**		Visual Association Test^§^	0**		N.A.
Visuoperception	Hooper visual integration	15					N.A.
Visuoconstruction	Rey’s complex figure (copy)^§^	13*	*z* = −2.9	Rey’s complex figure (copy)	11*	*z* = −3.2	29.8 (5.8)^b^
Attention	Stroop I^§^	3**	*z* = −0.5	TMT A^§^	4**	*z* = −0.6	12.9 (18.3)^c^
	Stroop interference score (III to II)^§^	42	*z* = 0.8				26.5 (18.9)^c^
	TMT A^§^	7*	z = −0.5				24.8 (32.5)^c^
	TMT interference score (B–A)^§^	24*	*z* = −0.3				32.2 (29.5)^c^
Executive Functioning	BADS key search^¥^	1**	*z* = −0.8				2.0 (1.2)^c^
				FDS Rating Scale	5/30**		N.A.
Intelligence	WAIS-III	70**	*z* = −2.4				104 (14)^e^
	WAIS-III verbal scale	74**	*z* = −2.4				107 (14)^e^
	WAIS-III performance scale	65**	*z* = −2.3				100 (15)^e^

N.A. = not available, *Below average performance compared to the healthy control norm group (≤16th percentile). **Impaired performance compared to the healthy control norm group (≤6th percentile). ^¶^Raw score; ^§^percentile score; ^¥^equivalent score (max 4).

*^a^A reference group of 20 patients with KS, with an average age of 57.6 (8.7) years (27)*.

*^b^A reference group of 16 patients with KS, with an average age of 58.9 (7.1) years (28)*.

*^c^A reference group of 19 patients with KS, with an average age of 58.8 (8.8) years (29)*.

*^d^A reference group of 20 patients with KS, with an average age of 59.7 (5.7) years (30)*.

*^e^A reference group of 30 patients with KS, with an average age of 60.5 (10.0) years (31)*.

## Results

### Observations and neuropsychological assessment on T1

Qualitative observations on T1 showed a consistent pattern of striking impairments. Mental confusion was reported, with a preference to keep asking for cigarettes, high distractibility, and disorganization of behavior. The patient would frequently walk away during a conversation. Her behavior appeared severely disorganized. While she was sitting behind a computer performing Solitaire, she would move her legs up and down vigorously, possibly as a symptom of late-stage WE (4). Moreover, she would walk into her room, undress, lay in her bed for just a couple of minutes. Hereafter she would dress herself, ask the nursing staff for cigarettes, smoke and walk into her room to undress and lay in her bed for just a couple of minutes, and dress again. During lunch and dinner time, she walked into the room and would just eat one bite and leave the room instantly. During the nights, she would ask the staff for food. According to nursing staff reports, she did not show evidence of positive nor negative emotions during the day.

The left column of Table 1 depicts the test scores on neuropsychological assessment after 4 months after admission to the hospital. Initially (T1), her performance on the neuropsychological assessment was consistent with a profile of cognitive deterioration, showing impaired performance on tests for attention, orientation, intelligence, word-fluency, working memory, long-term memory, construction, and executive functioning, compared to healthy participants. Importantly, the cognitive profile was severely affected by the attentional disorders, consistent with an acute WE. Neuropsychological performance ranged from moderate to severe impairment, with the exception of perceptual abilities. The reference groups of KS patients had an impaired performance on orientation, long-term memory, and executive functioning. On tasks intended to index orientation, word-fluency, working memory, visuoconstruction, and intelligence, the performance of the patient was more than 1 SD below the average score in the KS reference groups. This suggests that both the nature and extent of cognitive disorders in the case study were remarkably serious.

### Observations and neuropsychological assessment on T2

Qualitative observations of confused behavior lasted during her stay in our Korsakoff clinic without showing any signs of improvement or deterioration over time. The patient appeared as highly distractible if the team members were able to motivate her. She would frequently undress and redress herself in her room.

The right column of Table 1 depicts the test scores on neuropsychological assessment after 16 months after admission to the hospital. The pattern of cognitive functioning was consistent to the pattern of observed cognitive dysfunction at T1. The patient showed similar performance on the cognitive tasks: impaired performance on tests for orientation, intelligence, word-fluency, working memory, long-term memory, construction, attention, and executive functioning compared to healthy participants. Attentional problems were as striking as at T1. The cognitive pattern was stable compared to T1, whereas improvement in case of KS is to be expected based on the available literature (see Figure 1).

## Discussion

The patient in the present case report showed a distinctive chronic pattern of cognitive and neuropsychiatric impairments in the course of severe WE. Signs of confusion and incoherent behavior were evident and did not resolve in time. Cognitive disorders in attention, long-term memory, working memory, visuoconstruction, and word-fluency became apparent both after 4 and 16 months after admission to the hospital. Importantly, the attentional deficits severely affected the cognitive profile on both neuropsychological evaluations. Compared to a KS reference group, the patient showed a broader range of severe cognitive impairments. The patient was able to function independently up to the point that she developed WE. The cognitive and neuropsychiatric problems were in accordance to the acute symptoms of WE and did not improve over the course of her stay in the clinic, suggesting that her WE became chronic.

Wernicke’s encephalopathy is a life-threatening condition following acute thiamine deficiency (5). Whereas a large deal of research on WE has been devoted to successful treatment of WE in the acute phase, descriptions of cases where confusion does not improve rapidly are currently lacking. This is striking given that in clinical practice, WE is undertreated and patients are still admitted to general or psychiatric hospitals with prolonged states of confusion (13–15). Moreover, WE is potentially fatal in about 20% of the patients (8). The present case study suggests that WE can not only result in a life-threatening acute situation, but also in a chronic form of WE, incorporating a chronic state of confusion and disorganized behavior. The severity of the neuropsychiatric symptoms makes a patient in need of lifelong care.

A possible explanation for why the patient in the current case study developed a severe chronic pattern of cognitive problems could be the late hospital admission and inadequate treatment with thiamine replacement therapy. Although the patient was found lying on the floor with abundant symptoms of neurological disease (e.g., odd eye movements, visual hallucinations, incontinence), she was not admitted to the hospital in the first 5 days and received no treatment during this period. Since severe cognitive disorders are the consequence of untreated or under-treated thiamine deficiency, the patient should have received thiamine replacement therapy instantly (7). In the present case, WE was eventually diagnosed in the hospital, after which oral thiamine supplementation started. This time point of thiamine supplementation is regarded as a very late compensation for thiamine deficiency (13). Moreover, oral replacement therapy is less effective than parenteral replacement therapy (4).

Given that the prognosis of WE is known to depend on the speed of compensating the deficiency in thiamine (14) and the severe chronic cognitive disturbance associated with chronic WE observed in the present study, our results indicate that an active treatment policy is needed to avoid severe chronic neuropsychiatric symptoms. Current treatment standards suggest that parenteral (intravenous or intramuscular thiamine should be given 200 up to 500 mg three times daily until symptoms of acute WE resolute (32). In order to prevent severe and chronic cognitive disorders, physicians should have a high index of suspicion for WE and dose parenteral thiamine accordingly (7).

Although the patient in our case study had a history of alcohol abuse, the neurocognitive symptoms had an acute onset as reported in WE, which makes alcohol dementia an implausible explanation for the severe acute cognitive problems (33). Moreover, the cognitive problems remained stable over a period of 16 months, unlike progressive dementia. Dissimilar to KS, the patient had chronic problems in her attentional function, working memory, visuoconstruction, and word-fluency functions.

In conclusion, the current case study illustrates the cognitive and behavioral characteristics of a patient who was admitted to our clinic with untreated WE. Our description provides the first report on a patient with prolonged states of confusion following WE, suggesting that WE can become chronic in case of late thiamine replacement therapy.

## Conflict of Interest Statement

The authors declare that the research was conducted in the absence of any commercial or financial relationships that could be construed as a potential conflict of interest.
